# Role of Occupational Health Nurses in Mental Health Management: Insights From Supporting Overseas Employees

**DOI:** 10.7759/cureus.108490

**Published:** 2026-05-08

**Authors:** Hiroo Kaito, Hiromi Kuroishi

**Affiliations:** 1 Internal Medicine, Kaito Clinic, Tokyo, JPN; 2 Health Management, Mitsubishi Heavy Industries, Tokyo, JPN

**Keywords:** health support team, line-like relationships, mental health management, multidisciplinary collaboration, occupational health nurse, overseas employees, telemedicine

## Abstract

Background: The mental health of expatriate employees is a growing concern. While occupational health nurses (OHNs) are well-positioned to coordinate multidisciplinary support, their specific role remains underexplored in international literature.

Objective: This paper describes OHNs' role in coordinating multidisciplinary mental health support for expatriates. Based on 10 years of experience (2010-2020; N = 450) supporting Japanese engineers across eight international plant construction projects, we characterize this framework through three cases, exploring its feasibility and acceptability over effectiveness.

Methods: We used a descriptive, retrospective design based on routine occupational health work within a Japanese multinational engineering firm. Cases were purposively selected from eight overseas projects in five regional clusters: North Africa (Algeria), Sub-Saharan Africa (South Africa), Southeast Asia (Malaysia), Central Asia and the former Russian bloc (Uzbekistan, Turkmenistan, and Tatarstan), the Caribbean (Trinidad and Tobago), and South America (Chile). Data sources included health records, structured emails, semi-structured manager interviews, and field notes. Outcomes were evaluated using three objective indicators (safe repatriation, absence of significant residual harm, and absence of acute crises), key-informant ratings, and OHN observation notes. Newsletter feedback underwent inductive thematic analysis following Braun and Clarke’s framework. The study complied with the Declaration of Helsinki and received ethics approval from Kaito Clinic; individual consent was waived due to the use of de-identified retrospective records.

Results: Three illustrative cases are presented. Case 1 demonstrates the successful emergency aeromedical repatriation and return-to-work of an employee with acute psychiatric illness, aided by the OHN’s coordination and a pre-established referral pathway. Case 2 documents the reception of a multilingual monthly newsletter; 90 respondents (≈20% response rate) appreciated the culturally resonant content (55.6%), noted increased service awareness (44.4%), and felt connected to the home team (38.9%). Case 3 describes overseas site visits, rated favorably by 100% (22/22) of interviewed project managers. The three objective indicators were met across all cases, demonstrating practical feasibility and acceptability within this context rather than proven clinical effectiveness.

Conclusions: Positioning OHNs as coordinators within a multidisciplinary mental health support team is a feasible and well-accepted approach for Japanese technical employees on overseas assignments. Site visits, multilingual communication, and established referral pathways are practically valuable. Given the retrospective, single-organization design, these findings do not prove clinical effectiveness or generalizability. Further prospective, multi-organizational studies with validated outcome measures are needed to evaluate effectiveness and transferability to other contexts.

## Introduction

Employee mental health management has become an essential component of corporate strategy for maintaining productivity and organizational vitality [1]. The World Health Organization (WHO) estimates that approximately 15% of working-age adults experience a mental disorder at any given time, with depression and anxiety alone costing the global economy an estimated one trillion US dollars annually in lost productivity [2]. In response, the WHO released its first global guidelines on mental health at work in 2022, recommending organizational interventions, manager and worker training, and individual-level support as evidence-based strategies [2]. These developments have prompted diverse initiatives across organizations worldwide [1,3].

The recognition that work itself shapes the health of workers has deep historical roots, extending from Bernardino Ramazzini’s 18th-century *De Morbis Artificum Diatriba* [4], through the codification of industrial hygiene by Alice Hamilton in the early 20th century [5], to the postwar institutionalization of occupational medicine and occupational health nursing in many industrialized nations [6]. Across this trajectory, the discipline has progressively broadened from the prevention of physical injury and toxic exposure toward an integrated, biopsychosocial, and now whole-of-organization approach - the historical context within which the contemporary expansion of the occupational health nursing role should be understood.

While such initiatives are increasingly well-established for domestic workforces, mental health support for employees on overseas assignments presents distinctive challenges. Expatriates face heightened vulnerability to affective and adjustment disorders due to occupational anxiety, separation from home-country social networks, acculturation stress, and limited access to familiar healthcare systems [7,8]. Survey evidence suggests that a substantial proportion of expatriates do not consider mental health preparation before relocating, leaving many inadequately prepared for the psychological demands of expatriate life [9]. A non-trivial share of expatriates return prematurely from assignments due to adjustment difficulties, at considerable cost to both the individual and the organization [10]. Under the exceptional conditions imposed by the COVID-19 pandemic, evidence from international construction projects has further demonstrated that the mental health of expatriates depends not only on individual psychological resources but also on team-level and organizational-level support systems [11]. Accompanying spouses and partners likewise face significant stressors during expatriation, and the absence of targeted support interventions has repeatedly been identified as a primary and underreported cause of assignment failure [12].

Effective occupational health support requires seamless collaboration among multiple professionals, although the extent and quality of such collaboration vary substantially depending on the size and staffing of corporate health departments [3,13]. We posit that OHNs are well-suited to coordinate this multidisciplinary collaboration because of their extended contact time with employees, their broad foundational training in preventive medicine and public health nursing, and their capacity to build trusting relationships through ongoing engagement [14,15]. The American Association of Occupational Health Nurses has long recognized the role of OHNs in delivering comprehensive health and safety programs to worker populations [14]. More recently, the first international standard focused on psychological health, occupational safety, and well-being at work (ISO 45003:2021) has provided additional frameworks for managing psychosocial risks in the workplace [16]. International occupational health nursing competency frameworks similarly emphasize an expanded role in psychosocial coordination and cross-cultural practice [15,17].

Within this broader landscape, the present paper describes one organization’s practice experience in operating an OHN-coordinated mental health support framework for overseas employees. We focus particularly on the value of continuous, “line-like” engagement of OHNs with employees - an ongoing, longitudinal relationship that extends across pre-deployment, deployment, and post-deployment phases - as opposed to episodic, “point-like” interactions. Continuity of engagement enables the detection of subtle changes in employee well-being that would be invisible in isolated encounters. In keeping with the descriptive nature of this report, we present the framework and three illustrative cases as practice experience rather than as evidence of effectiveness.

Study objectives

This paper aims to describe the role of OHNs as coordinators of a multidisciplinary mental health support team for employees on overseas assignments. The framework was operated continuously over ten fiscal years and supported a cumulative cohort deployed across eight international plant construction projects; the detailed scope and demographics are presented in the Methods section.

The objectives are organized into one primary aim and two secondary aims.

Primary Aim

This study aimed to describe the structure and day-to-day operation of an OHN-coordinated multidisciplinary mental health support framework deployed across eight overseas plant construction projects.

Secondary Aims

This study also aimed (1) to illustrate the practical implementation of the framework through three representative cases (emergency psychiatric repatriation, multilingual health newsletter, and overseas site visits) and (2) to explore, in a hypothesis-generating rather than confirmatory manner, the feasibility and acceptability of this coordinated framework using a mixed evaluation framework consistent with the practice-based context.

Consistent with these aims, the present report does not seek to demonstrate clinical effectiveness, comparative superiority, or generalizable benefit.

## Materials and methods

Study design and reporting standards

This study employs a descriptive, retrospective, practice-based design drawing on the authors’ routine professional experience providing occupational health support to overseas employees within a Japan-headquartered multinational engineering organization with globally distributed operations. The design is explicitly descriptive: it represents a reflective account of an established occupational health support system rather than a prospective interventional or controlled study. There was no a priori research protocol, no control or comparison group, no randomization, no pre/post comparison on the full deployed population, and no application of validated quantitative mental health outcome instruments. We followed reporting principles broadly aligned with the Standards for Reporting Qualitative Research (SRQR) and the relevant elements of the Consolidated Criteria for Reporting Qualitative Research (COREQ) where applicable to a practice-based report.

Study setting and period

The study setting is a Japan-headquartered multinational engineering organization that deploys home-country technical employees to overseas plant-construction sites, including locations in regions with limited medical infrastructure. The OHN-coordinated occupational health support framework described here has been in continuous operation since April 2010. The observation period covered in this manuscript runs from April 2010 to March 2020 (10 fiscal years). All data were drawn from routinely collected occupational health records, organizational program documentation, structured email correspondence, semi-structured manager interviews conducted as part of routine site visits, and the authors’ own field notes from on-site rounds. No additional prospective data collection was undertaken for this report.

Ethical approval and consent

The study was conducted in accordance with the Declaration of Helsinki (2013 revision). The study was reviewed and approved by the institutional ethics review committee of Kaito Clinic (dated March 22, 2025), with appropriate documentation of the approval and the corresponding ethical review process maintained on file. Because the study constituted a retrospective analysis of de-identified, routinely collected occupational health records and field notes, the requirement for individual prospective informed consent was waived. All identifying information was removed from case descriptions; details that might permit individual or project-level identification (specific dates, hospitals, assignment durations, and personal characteristics) have been altered or aggregated as necessary to protect confidentiality.

Case selection and inclusion criteria

Overseas plant construction projects within this organization occur at a frequency of approximately one new project per fiscal year. For this study, cases were purposively selected from among all eight plant construction projects in which the authors’ occupational health team had direct medical involvement during the observation period, including on-site health support, remote medical consultation, and emergency coordination. The inclusion criterion was the presence of documented occupational health team engagement: specifically, projects in which the OHN-coordinated multidisciplinary team provided medical support services. The three cases presented (Cases 1-3) were further selected on the following pre-specified criteria: (a) representativeness of one of the three principal modes of OHN coordination (acute crisis response, asynchronous health communication, and on-site face-to-face support), (b) availability of sufficient documentation in the routine records to permit a structured account, and (c) feasibility of de-identified presentation.

We explicitly acknowledge that this purposive selection introduces selection bias whose probable direction is toward favourable outcomes. Cases were drawn exclusively from projects in which the OHN-coordinated team was directly and actively involved; projects in which intensive health support was logistically infeasible (for instance, because of the very short duration of the project, restricted access to the worksite, or the absence of an on-site safety and health structure) were necessarily excluded. Such excluded projects might plausibly have experienced different and, in some respects, worse mental health trajectories. The findings should therefore be interpreted as illustrative of what is possible when a multidisciplinary OHN-coordinated team is fully resourced and operationally embedded, rather than as representative of the average overseas project across the industry.

To help readers assess representativeness, we offer the following contextual comparison between the included and the broader set of non-included or partially included engagements over the same period, while acknowledging that exact contemporaneous data on the latter are inevitably incomplete given the practice-based nature of the work. The eight included projects were characterized, broadly, by (i) longer assignment durations (typically multi-year), (ii) on-site safety and health structures sufficient to host the visiting occupational health team, (iii) project leadership willing to allocate operational time for in-person consultations and manager interviews, and (iv) corporate budgetary support for annual circuit visits and multilingual newsletter dissemination. Engagements that fell outside this report, such as very short technical dispatches, sub-contracted assignments to remote sites without a dedicated safety and health coordinator, and projects in which the occupational health team was consulted only intermittently or only by remote channels, generally lacked one or more of these enabling conditions. It is plausible, although not directly demonstrable from the present dataset, that such engagements were associated with thinner documentation, lower-intensity follow-up, and a higher likelihood that any incipient mental health concerns went unrecognized or unrecorded. The probable direction of the resulting selection bias is therefore twofold: First, the included cases are likely to over-represent settings in which the OHN-coordinated framework could be most fully implemented. Second, the favorable acceptability and feasibility findings reported here may not extrapolate to lean deployment contexts where similar structural support is unavailable. We have refrained from constructing a formal numerical comparison between included and non-included engagements because the underlying records for the latter are heterogeneous and incomplete. Readers are accordingly invited to treat the present findings as a best-case-scenario description of what an adequately resourced OHN-coordinated framework can look like, rather than as a representative cross-section of overseas plant-construction work as a whole.

Scope of the program

Eight overseas plant construction projects were supported by the OHN-coordinated team during the observation period. The projects were located in eight countries spanning five regional clusters: North Africa (Algeria), Sub-Saharan Africa (South Africa), Southeast Asia (Malaysia), Central Asia and the former Russian bloc (Uzbekistan, Turkmenistan, and Tatarstan), the Caribbean (Trinidad and Tobago), and South America (Chile). Worksites were typically located outside major urban centers and included desert, high-altitude, and extremely cold environments with markedly limited local medical infrastructure. A total of 450 home country-dispatched Japanese technical employees were covered across the eight projects (mean 56.3 per project; median 55; range 40-90). The per-project headcounts are presented in Table 1. In addition to these home country staff, each project team incorporated locally recruited technical personnel who comprised approximately half of the overall on-site project workforce under contractual arrangements negotiated at each construction site. In accordance with corporate policy and the scope of the occupational health team’s mandate, the on-site health support activities described here focused exclusively on the home country-dispatched Japanese technical employees.

**Table 1:** Geographic coverage Geographic coverage and workforce of the eight overseas plant construction projects covered by the OHN-coordinated support framework (cumulative N = 450 home country-dispatched Japanese technical employees over the observation period).

No.	Country	Regional cluster	Japanese technical staff (n)
1	Algeria	North Africa	90
2	South Africa	Sub-Saharan Africa	60
3	Malaysia	Southeast Asia	50
4	Uzbekistan	Central Asia	60
5	Turkmenistan	Central Asia	50
6	Tatarstan (Russian Federation)	Former Russian bloc	40
7	Trinidad and Tobago	Caribbean	40
8	Chile	South America	60
Total	Eight countries	Five regional clusters	450

Data collection methods

Observations and data were collected through four principal methods. Each method is described in detail below to support transparency and partial reproducibility. We emphasize, however, that this report describes routine occupational health practice rather than a prospective research protocol. The absolute reproducibility of context-dependent practice judgments is inherently limited.

Method 1: On-Site Face-to-Face Individual Consultations

During scheduled annual on-site circuit visits (in principle, one visit per project per fiscal year, supplemented by two to three additional visits per year following on-site incidents), the occupational health team conducted face-to-face individual consultations with all home country-dispatched Japanese technical employees present at the site. Each consultation lasted 30-60 minutes and was conducted in Japanese (the employees’ native language) in a private room at the site clinic or, where no clinic was available, in an equivalent private space identified jointly with the project manager. Consultations followed a semi-structured guide covering eight standard topic areas: (i) physical health status (sleep, appetite, weight, gastrointestinal and musculoskeletal complaints, and climate-related symptoms); (ii) mental health status (mood, anxiety, irritability, hopelessness, and suicidal ideation if appropriate); (iii) workplace conditions (working hours, workload, inter-team relationships, and perceived support from supervisors); (iv) dormitory and daily living circumstances (food, water, hygiene, and leisure); (v) climate, infrastructure, and security; (vi) access to and prior use of local medical resources; (vii) family and home-country contact (frequency and perceived adequacy); and (viii) overall self-rated well-being. The standard guide was developed by the OHN and the occupational physician based on routine clinical practice and was iteratively refined across the observation period. It was not derived from a single published, validated instrument. During each consultation, the OHN performed a structured observational assessment of employee affect and demeanour, for example, facial expression, eye contact, posture, speech rate and prosody, and the presence or absence of visible signs of distress, fatigue, or anxiety, analogous to a simplified faces-scale clinical observation. Observational notes were recorded in the OHN’s consultation notebook in narrative form and subsequently transferred into the case record.

Method 2: Structured Email Follow-Up Correspondence

For employees identified during on-site consultations as requiring further attention (any of subjectively reported persistent low mood or sleep disturbance, OHN-rated abnormal affect, physician concern, and employee request for additional contact), structured email follow-up correspondence was initiated from the home country following the team’s return. Follow-up was conducted in Japanese on a multiple-round basis, in principle every two to four weeks, and continued until the original concern was clinically resolved, the employee returned to the home country, or specialist referral had been securely established. The standard email template prompted updates on the same eight topic areas covered in the on-site consultation, plus a free-text section for any new concerns. Replies were received as Japanese text and were filed in the case record together with the OHN’s response and any escalation actions.

Method 3: Semi-structured Interviews With Project Managers

During each circuit visit, semi-structured interviews were conducted with project managers and senior on-site personnel. We aimed to interview every available manager; in practice, approximately 70% of on-site project managers were available within the visit window and participated. Across the eight projects and the observation period, a cumulative total of 22 individual project managers and senior on-site supervisors were interviewed (denominator described under the Outcome Evaluation Framework). Each interview lasted approximately 60 minutes (range 60-90 minutes) and covered (a) the manager’s overall observation of employee well-being on the project; (b) any episodes of physical or mental-health concern of which the manager was aware; (c) the manager’s appraisal of the on-site health-support activities (consultations, lectures, environmental survey, newsletter, and follow-up correspondence); (d) suggestions for improvement; and (e) the manager’s view on whether the programme should continue at the next visit cycle. Interviews were conducted in Japanese. Given the operational and managerial context, audio recording was not feasible, and interviews were not transcribed verbatim. Detailed contemporaneous handwritten notes were taken by the OHN and were typed up into a structured visit report.

Method 4: Post-visit Corporate Report and Feedback

After the team’s return from each circuit visit, a full site-visit report (approximately 100 pages per project, including narrative summary, anonymized case observations, environmental survey results, manager-interview summary, and recommended follow-up actions) was submitted to senior project management and to corporate leadership at the head office. Feedback was received from approximately 30% of senior recipients in the form of written comments, follow-up meeting requests, or implementation decisions. A multilingual monthly health newsletter (the Minatomirai Health Newsletter) was disseminated electronically to the entire deployed cohort throughout the observation period.

Data management and synthesis

Information from all four sources was systematically compiled by the OHN within the corporate occupational health electronic record system, cross-referenced where possible, and organized into per-project case records. Data were managed in standard Microsoft Excel spreadsheets (Microsoft Corp., USA); no specialized qualitative data-analysis software (such as NVivo or MAXQDA) was used. All identifying information was removed before analysis; the OHN and the occupational physician maintained the only access to the linked dataset, and the de-identified analytical dataset used for this manuscript is held on a password-protected institutional server. All data collection was conducted as part of routine occupational health practice and did not constitute a prospective research protocol. We acknowledge that strict scientific reproducibility in an external setting is therefore inherently limited. The methods described here are intended to enable conceptual replication and adaptation rather than literal external reproduction.

Establishment of the multidisciplinary health support team

A comprehensive, multidisciplinary health support team was established, comprising the following five professional roles: occupational physician, psychiatrist, OHN, clinical psychologist, and safety and health manager. This composition was designed to provide holistic coverage across preventive, diagnostic, therapeutic, and administrative dimensions of employee health management. The team structure reflects best practices recommended by the WHO and the International Labour Organization (ILO), which emphasize that workplace mental health interventions should encompass organizational, managerial, and individual levels [18].

Selection of the OHN as team coordinator

To facilitate smooth interdisciplinary collaboration, the OHN was designated as the team coordinator. This selection was based on three principal considerations: (a) the OHN’s broad foundational knowledge spanning preventive medicine, public health nursing, and travel medicine; (b) the OHN’s frequency of direct interaction with employees, which substantially exceeds that of other team members; and (c) the OHN’s demonstrated ability to establish and maintain trust-based, continuous (“line-like”) relationships with employees across the entire arc of their overseas assignments. The coordinator role encompasses initial triage, inter-professional communication, information synthesis, follow-up scheduling, and longitudinal case tracking.

Definition of OHN Duties

The specific duties assigned to the OHN within this framework were organized into six domains, as detailed in Table 2.

**Table 2:** Specific duties of the occupational health nurse within the multidisciplinary support team, organized into six functional domains

Role category	Specific duties
Preventive medicine	Conducting health examinations; follow-up actions based on examination results (recommendations for re-examination, detailed examinations, referrals to medical institutions).
Health education	Providing nutritional guidance, exercise instruction, and practical advice for improving lifestyle habits in daily life.
Emergency medical care	In coordination with on-site safety and health staff, actively supporting the prompt early arrangement of visits to local clinics or hospitals for employees requiring urgent attention.
Travel medicine	Offering pre- and post-deployment health consultations to overseas-assigned employees and accompanying families; providing updated global health information on infectious diseases, vaccines, and region-specific risks.
Mental health support	Serving as the primary consultation desk for general inquiries and mental-health concerns of overseas-assigned employees and accompanying families, with triage and referral to clinical psychologists and psychiatrists as appropriate.
Global health information	Providing continuously updated global health information on infectious diseases and vaccines, with particular attention to destination-specific risks.

Practical methods for early detection

The OHN served as the pivotal coordinator within the health support team for facilitating the early detection of mental-health concerns. The detection methodology operated through a two-step process, as described in Table 3.

**Table 3:** Comprehensive multifaceted approach to supporting employees’ mental health within the OHN-coordinated framework OHN: occupational health nurse

Category	Support type	Details
Individual support	Continuous counselling	Ongoing counselling and consultations provided by the OHN, with specialist referral to clinical psychologists and psychiatrists as indicated.
Proactive measures	Self-discovery and voluntary consultation	Implementation of proactive methods encouraging employees to identify their own mental-health concerns; voluntary access to in-house psychiatrists and clinical psychologists; OHN facilitation of communication between specialists and the occupational physician.
Collective support	Awareness campaigns and seminars	Creation and dissemination of original educational materials on physical and mental health via company intranet and email; regular in-person health seminars targeting high-risk groups.
Unique overseas support	Site visits and environmental surveys	On-site medical consultations at overseas work locations; individual and group mental-health assessments during regular visits; provision of local medical-facility information; workplace and hygiene environment surveys conducted jointly by the occupational physician and OHN.

Multifaceted approach to mental health support

A comprehensive, multifaceted approach was implemented, encompassing four domains of support, as described in Table 4.

**Table 4:** Comprehensive multifaceted approach to supporting employees’ mental health within the OHN-coordinated framework OHN: occupational health nurse

Category	Support type	Details
Individual support	Continuous counselling	Ongoing counselling and consultations provided by the OHN, with specialist referral to clinical psychologists and psychiatrists as indicated.
Proactive measures	Self-discovery and voluntary consultation	Implementation of proactive methods encouraging employees to identify their own mental-health concerns; voluntary access to in-house psychiatrists and clinical psychologists; OHN facilitation of communication between specialists and the occupational physician.
Collective support	Awareness campaigns and seminars	Creation and dissemination of original educational materials on physical and mental health via company intranet and email; regular in-person health seminars targeting high-risk groups.
Unique overseas support	Site visits and environmental surveys	On-site medical consultations at overseas work locations; individual and group mental-health assessments during regular visits; provision of local medical-facility information; workplace and hygiene environment surveys conducted jointly by the occupational physician and OHN.

Outcome evaluation framework

Evaluation of the OHN-coordinated support framework employed a mixed approach combining objective outcome indicators with semi-quantitative key-informant ratings and qualitative observational appraisals, consistent with the descriptive and retrospective nature of this study. No validated standardized outcome instruments (such as the Patient Health Questionnaire-9 (PHQ-9) for depression, the Generalized Anxiety Disorder-7 (GAD-7) for anxiety, or the WHO-Five Well-Being Index (WHO-5)) were applied prospectively, because the support activities were implemented within routine occupational health practice rather than as a controlled research protocol. We address this limitation explicitly in the Limitations section.

Operational definitions of objective indicators

Three objective outcome indicators were defined a priori for the present manuscript, based on what was reliably and verifiably documented in the routine occupational health record:

Safe Repatriation

For any employee for whom medical evacuation was initiated, the indicator was met if the employee was successfully transported from the overseas worksite to a designated home-country medical facility without an in-transit medical event (defined as any unplanned medical intervention en route, in-flight diversion for the patient’s condition, or arrival in a clinically deteriorated state requiring resuscitation).

Absence of Clinically Significant Residual Occupational Health Harm

For any employee involved in a documented mental health event, the indicator was met if, at the most recent occupational health review documented within 12 months of the event, the employee had no documented persisting psychiatric diagnosis attributable to the deployment-related event, no documented inability to perform pre-event duties, and no documented prescription of long-term psychotropic medication initiated and continued for the deployment-related condition.

Absence of Acute Mental Health Crises

At the project level, the indicator was met if, during the observation window of the project, no acute psychiatric event was documented requiring emergency aeromedical evacuation, involuntary admission, or active suicide prevention intervention. The denominator for project-level analysis is the eight projects.

These indicators are deliberately binary and crude. We do not claim that they measure mental health improvement at the population level. They reflect what could be reliably extracted from routine records.

Semi-quantitative key informant appraisals

Semi-quantitative key informant appraisals were obtained retrospectively from a purposively selected subset of project personnel who had directly observed or received the occupational health services. Informants comprised 22 individuals across the eight projects: project managers (n = 12), senior site supervisors (n = 6), and safety and health representatives (n = 4). Informants were asked a single standardized question for each support activity: “Considering the [activity] as a whole, how would you characterize its overall value to the project: unfavourable, neutral, or favorable?” This three-category ordinal scale was adopted in preference to a continuous numerical scale because the small purposively selected informant sample (n = 22) did not support meaningful calculation of summary statistics, and because a coarser categorical scale was felt to better suit the on-site interview context. We acknowledge that ordinal data of this kind risk an acquiescence bias and a positive courtesy bias in an interview conducted by the programme providers; this is discussed under Limitations. Where reported in the Results, the numerator and denominator are provided in full (e.g., 22/22 favorable).

Observational affect assessments

Observational affect assessments were performed by the OHN during all face-to-face consultations at overseas sites. Consistent with clinical nursing practice, the OHN systematically noted observable indicators of employee psychological state, including facial expression, eye contact, posture, general demeanour, speech rate and prosody, verbalized concerns, and apparent affect, analogous to a simplified face-scale observation. These observational impressions were recorded in narrative form in the OHN’s consultation notebook and contributed to the overall longitudinal appraisal of employee well-being. They do not constitute validated psychometric measurements. Their value lies in supporting the OHN’s longitudinal clinical judgment rather than in producing aggregate quantitative data, and we report them as such.

Reporting of approximate values

Several aggregate proportions reported in the Results section (notably employee recognition of the site-visit program and the proportion of employees reporting a strengthened sense of connection with the home country team) are approximations derived from routine clinical records and field notes rather than from a formal sampling frame. Wherever exact numerators and denominators could be retrieved from the routine records, they are reported (e.g., 22/22 favorable key informant ratings; 90/450 returned newsletter questionnaires; 8/8 project leaderships requesting continuation). For values reported as approximate (denoted by the symbol ≈ or by the phrasing “approximately”), we explicitly note that the value was derived from routine clinical records and field notes. We have used a single, consistent format throughout the manuscript.

To improve clarity and interpretability, and in direct response to reviewer feedback on this point, we summarize here the practical derivation of each approximated value, while acknowledging openly that some residual imprecision is unavoidable given the practice-based nature of the records. The figure of approximately >90% employee recognition of the OHN-coordinated site-visit program was derived qualitatively from the on-site interview log and the post-return health examination notes covering the cumulative deployed cohort over the observation period. Specifically, during on-site individual consultations and the routinely scheduled post-return health examinations, the OHN documented whether each employee was already aware of the programme and its activities, and the documented proportion was consistently above nine in 10 across projects. The figure of approximately 95% of employees reporting a strengthened sense of connection with the home-country team was derived from the same combined documentation (on-site interviews plus post-return health examinations), where employees were asked an open-ended question about their perceived connection with home country health support and the OHN classified the recorded response, post hoc, as “strengthened,” “unchanged,” or “weakened." Values clustered consistently in the “strengthened” category at approximately the level reported. The figure of approximately 70% of on-site project managers interviewed per visit reflects the realized interview rate per visit window, recorded in the circuit-visit log. The exact denominator varied with on-site rotation and could not be reconstructed retrospectively to the level of a fixed eligible cohort. The figure of approximately 30% written response rate from senior corporate recipients of the post-visit report was derived from the feedback log maintained by the head-office occupational health team. The frequency of supplementary visits (two to three per year per project following on-site incidents) was derived from the same circuit visit log. We have aimed for a single, consistent format throughout the manuscript: exact ratios are presented as numerator/denominator with associated percentages; approximations are marked with ≈ or with the word “approximately,” and the data source for each approximation is named at the point of first reporting and re-stated in the relevant table footnote. We have not used any approximated value to support an inferential statistical claim, and we explicitly flag in the Limitations section that these approximations preclude precise re-analysis.

Thematic analysis procedure

For Case 2, structured email feedback questionnaires were distributed to all deployed employees after each newsletter edition. Over the 12 monthly editions analyzed for this report, a cumulative total of 90 completed questionnaire responses were returned. The response rate is reported as approximately 20% because the denominator is itself approximate: the cumulative head count of 450 reflects deployments over the eight projects across the observation window, while the actual responding-eligible population in any given month was somewhat smaller, depending on which employees were on assignment, on home leave, or had completed the project rotation.

Responses were analyzed using an inductive thematic analysis approach broadly consistent with Braun and Clarke’s six-phase framework [19]. The analysis was conducted by two reviewers: the OHN (an experienced OHN) and the occupational physician. Analysis steps were as follows: (i) both reviewers independently read all 90 responses to familiarize themselves with the data. (ii) Each reviewer generated initial open codes line by line, without a pre-specified coding frame. (iii) Codes were then independently clustered by each reviewer into candidate themes. (iv) Candidate themes from the two reviewers were compared, areas of disagreement were discussed in iterative consensus meetings, and a consolidated theme set was agreed upon. (v) The consolidated themes were reviewed against the original responses to confirm that each theme was supported by recurrent, distinct content. (vi) Final themes were named and defined.

An illustrative excerpt of the coding framework is provided in Table 5 to support transparency. Initial open codes (left column) were inductively generated from the response text and were then iteratively clustered into the broader themes finally retained (right column). The full coding ledger is held by the corresponding author and is available on reasonable request.

**Table 5:** Illustrative excerpt of the inductive coding framework used for the thematic analysis of newsletter feedback (Case 2) Initial open codes were generated independently by two reviewers and were iteratively clustered into the three consolidated themes retained for analysis.

Initial open codes (illustrative examples)	Final consolidated theme
"Felt encouraged by the essay"; "Stories of inspirational figures gave me strength"; "The cultural reflections lifted my spirits"; "More than just medical information".	Theme A: Appreciation for motivational and culturally resonant content
"Now I know whom to contact in HQ"; "Did not realize a psychiatrist was available"; "Will use the consultation desk next time"; "Helpful to know about the in-house team".	Theme B: Increased awareness of available health support services
"Reminds me HQ has not forgotten us"; "Feel less isolated"; "Newsletter is a thread back home"; "Reassuring to receive every month".	Theme C: Sustained sense of connection with home-country health support team

Inter-reviewer agreement was assessed through structured consensus discussion rather than through formal statistical metrics (such as Cohen’s kappa or percent agreement on a fixed code list). Several factors made a formal statistical assessment of agreement inappropriate in this practice-based context: the dataset was small, and the coding was inductive (so the candidate code list itself was iteratively co-developed rather than fixed in advance), the two reviewers were the same individuals who had collaborated longitudinally on the programme, and the analysis was conducted retrospectively from routinely collected feedback rather than as a planned qualitative study. To enhance the validity of the analysis nonetheless, the two reviewers (a) independently completed steps (i)-(iii) before any joint discussion, (b) documented their independent code lists and candidate themes in writing before consensus meetings, (c) resolved each point of disagreement explicitly with reference back to the original response text, and (d) retained only those themes that were independently identified by both reviewers and that were reflected in at least 10% of responses (≥9 of 90 responses). Three themes met these criteria and are reported in the Results.

In direct response to reviewer feedback requesting a fuller account of how reviewer agreement was assessed beyond consensus discussion, we additionally describe here the procedural safeguards adopted to mitigate single-team bias as far as the practice-based setting allowed. First, prior to any joint meeting, each reviewer independently produced a written list of open codes and a written list of candidate themes; these two written documents were then compared side by side at the consensus meeting, with explicit identification of (a) themes independently named by both reviewers, (b) themes named by only one reviewer, and (c) divergent boundary placements between candidate themes. The proportion of candidate themes that were independently identified by both reviewers (group A) was qualitatively high in our experience, although we did not compute this as a formal numerical statistic because the inductive co-development of the code list precluded a stable shared denominator. Second, candidate themes named by only one reviewer (group B) were not silently dropped: each was discussed against the underlying response excerpts, and was either incorporated into a re-defined consolidated theme or explicitly set aside with a documented rationale. Third, for the three themes ultimately retained, both reviewers re-read every response text passage that had been mapped to that theme and independently confirmed that the mapping remained appropriate after consolidation; any residual disagreement at this stage led to either re-wording of the theme definition or removal of the borderline excerpt from the supporting evidence base. Fourth, the analytical decisions taken at each consensus meeting were recorded in a written audit memorandum (date, points discussed, resolution, and any remaining uncertainty), so that the chain of reasoning would be auditable on later review. We acknowledge frankly that these procedural safeguards do not substitute for an independent third coder or for a numerically expressed inter-rater statistic, and that the small inductive dataset and the longstanding collaboration of the two reviewers introduce an irreducible degree of shared-perspective bias. We have therefore presented the thematic findings in the Results as descriptive and exploratory only, and we discuss these constraints further in the Limitations section.

The structure of the three retained themes (presented in Table 5) can be summarized concisely so that readers may assess the coding logic. Theme A captured responses that connected the newsletter content to subjective experiences of motivation, encouragement, or cultural resonance - for example, references to the inspirational essays, to philosophical or biographical content, or to the sense that the newsletter contained “more than just medical information.” Theme B captured responses that referenced concrete recognition of, or intent to use, the in-house health-support services - for example, references to the consultation desk, the in-house psychiatrist or clinical psychologist, or the home-country occupational health team. Theme C captured responses that explicitly named a sense of continued relational link with the home-country team - for example, references to feeling “less isolated,” to the regularity of monthly contact, or to the newsletter as a perceived “thread” back home. The boundary between Themes B and C was the most discussed during consensus: Theme B was retained for content centred on awareness of services as an organisational resource, while Theme C was retained for content centred on the affective experience of relational continuity. Excerpts that referenced both elements were coded under both themes, in keeping with the non-mutually-exclusive coding rule stated in the Results.

Data management and coding were conducted manually using standard spreadsheet software (Microsoft Excel). No specialized qualitative data-analysis software (such as NVivo or MAXQDA) was used. We acknowledge that the use of dedicated qualitative software might have facilitated audit-trail documentation; in our practice-based setting, the use of spreadsheet software was judged appropriate to the scale of the data. Specifically, with a total of 90 short-form responses analysed inductively by two reviewers who had a longstanding shared working knowledge of the deployed cohort, the principal value that dedicated qualitative software would have added, namely, structured query and retrieval across a large corpus, formal cross-coder reliability calculation against a fixed code list, and systematic version control of an evolving codebook, was not, in our judgement, proportionate to the burden of data preparation and software learning at the moment when this retrospective analysis was undertaken. We have nonetheless preserved the manual coding worksheets, the per-reviewer initial code lists, the audit memoranda from each consensus meeting, and the final theme-mapped excerpt set in their original spreadsheet form, and these remain available, in de-identified form, on reasonable request to the corresponding author. We note transparently that, were a comparable analysis to be undertaken prospectively today on a larger or more heterogeneous dataset, the use of dedicated qualitative analysis software would be preferable.

## Results

Quantitative overview of the program

Before presenting the illustrative cases, we summarize the overall scale and intervention profile of the OHN-coordinated program. A consolidated numerical overview is provided in Table 6. Where values are reported as approximate, the data source (routine clinical records and field notes) is explicitly noted.

**Table 6:** Quantitative overview of the OHN-coordinated overseas health-support program (April 2010-March 2020) Values reported as approximate are explicitly attributed to routine clinical records and field notes. OHN: occupational health nurse

Program dimension	Value (and source, where approximate)
Number of overseas plant-construction projects supported	8 (exact)
Countries covered	8 (Algeria, South Africa, Malaysia, Uzbekistan, Turkmenistan, Tatarstan, Trinidad and Tobago, and Chile)
Regional clusters	5 (North Africa, Sub-Saharan Africa, Southeast Asia, Central Asia/former Russian bloc, Latin America and Caribbean)
Total home-country-dispatched Japanese technical employees covered	450 (exact, cumulative across the eight projects; mean 56.3, median 55, range 40–90 per project)
Frequency of on-site circuit visits per project	1 scheduled visit per fiscal year, plus two to three supplementary visits per year following on-site incidents (approximate; from the circuit-visit log)
Coverage of individual face-to-face consultations during each circuit visit	All on-site Japanese technical employees invited in principle; 30–60 minutes per consultation (per OHN consultation notebook)
Project managers and senior on-site personnel interviewed (cumulative across the observation period)	22 individuals interviewed in total (exact; from interview log): project managers n = 12; senior site supervisors n = 6; safety-and-health representatives n = 4. Each interview is ≈60 minutes (range 60–90 minutes).
Approximate proportion of on-site project managers interviewed per visit	Approximately 70% (estimate from circuit-visit log; exact denominator varied with on-site rotation)
Post-visit written report to corporate leadership	Approximately 100 pages per project (from corporate report archive); written feedback received from approximately 30% of senior recipients (estimate from feedback log)
Monthly multilingual health newsletter	Distributed to the entire deployed cohort throughout the observation period (12 monthly editions analysed for this report)
Newsletter feedback questionnaires returned	90 completed responses cumulatively over the 12 analyzed editions (exact); response rate ≈ 20% relative to the cumulative deployed population.
Structured email follow-up with employees identified as requiring attention	Multiple rounds per individual (approximately every two to four weeks), continued until concern resolved (exact per-individual count varied)
Intervention types	Annual on-site circuit visits with individual consultations, group health lectures, environmental surveys, emergency evacuation coordination, post-visit email follow-up, monthly newsletter, and corporate feedback reporting

Across the eight projects, the number of on-site individual consultations conducted during each scheduled annual circuit visit approximated the total on-site headcount of Japanese technical staff (target coverage 100%); the actual realized coverage in any given visit depended on operational availability at the time of the visit but consistently reached the large majority of deployed staff. Post-visit structured email follow-up was initiated with every employee identified by the OHN as requiring continued attention; exact counts varied by project and year.

On the basis of this multifaceted support framework, the following three illustrative cases illustrate distinct dimensions of the OHN-coordinated support system. Findings are presented as documented observations; interpretive commentary is reserved for the Discussion section.

Case 1: Emergency Aeromedical Repatriation and Return to Work

An employee stationed in a region of Asia with limited medical resources experienced an acute onset of psychiatric illness. The OHN received the initial emergency communication from overseas and initiated the following documented sequence of actions: (a) immediate remote counselling support was provided to the employee and on-site colleagues; (b) medical evacuation to the home country was arranged within 48 hours of initial contact; and (c) referral to the team psychiatrist, who was affiliated with a hospital equipped for emergency psychiatric admission, was completed on the day of repatriation. This pre-established referral pathway was a direct structural product of the team’s planning.

Regarding objective outcome indicators, the employee was safely repatriated without an in-transit medical event (indicator (a) met), received inpatient psychiatric treatment, achieved clinical recovery, and successfully returned to pre-event duties within the 12-month follow-up window with no documented persistent psychiatric diagnosis or long-term psychotropic medication attributable to the deployment-related event (indicator (b) met). In the post-project semi-quantitative appraisal, the project manager who served as the key informant for this case (one of one informant for this event) categorized the overall emergency-health-support response as “favourable” (1/1, 100%), specifically noting the value of the pre-established psychiatric referral pathway. We note explicitly that the appraisal was provided by a single informant (the project manager directly involved in coordinating the evacuation); a single favorable appraisal demonstrates that the system worked in this specific instance and is not generalizable to assertions about overall effectiveness.

Case 2: Multilingual Health Newsletter and Awareness Campaign

A periodic multilingual health newsletter (the Minatomirai Health Newsletter) was developed and distributed each month to the entire deployed cohort. The newsletter combined practical medical information with culturally resonant content, including biographical essays on inspirational figures and reflective writing connecting biomedical topics to broader cultural and philosophical themes. Representative pages of the English-language edition are presented in Appendices A-C. Employee feedback was collected through structured email questionnaires distributed following each monthly edition.

Across 12 monthly editions analyzed for this report, a cumulative 90 completed questionnaire responses were received (90/450 cumulative response rate ≈20% relative to the cumulative deployed population; the denominator is approximate because the responding eligible population varied month to month, as noted under Methods). Responses were subjected to the inductive thematic-analysis procedure described in the Methods section, with two independent coders, an iterative consensus process, manual coding using standard spreadsheet software, and a retention threshold of 10% of responses per theme.

Three recurrent themes met the pre-specified criteria and were retained: (a) appreciation for motivational and culturally resonant content, endorsed in 50 of 90 responses (55.6%); (b) increased awareness of available health-support services, endorsed in 40 of 90 responses (44.4%); and (c) sustained sense of connection with the home-country health support team, endorsed in 35 of 90 responses (38.9%). Themes were not mutually exclusive (a single response could endorse more than one theme). Semi-quantitative key-informant appraisals of the newsletter program were obtained from all 22 of 22 interviewed key informants across the eight projects (22/22, 100% completion of the appraisal question). All 22 informants categorized the newsletter program as “favorable” (22/22, 100%); no informant rated the program as “neutral” or “unfavorable.” We acknowledge explicitly that this 100% favorable rating, obtained from a small purposively selected informant group interviewed by the program providers themselves, is best interpreted as evidence of acceptability rather than as evidence of effectiveness, and may reflect courtesy or acquiescence bias. Numerical summaries are provided in Table 7.

**Table 7:** Interpretation of thematic feedback and key informant appraisals in the multilingual health newsletter program (Case 2) Themes were non-exclusive and were retained when endorsed by at least 10% of respondents and independently identified by both reviewers. The 100% favourable key-informant rating reflects a small, purposively selected, provider-administered interview and should be interpreted as evidence of acceptability rather than effectiveness.

Theme / rating category	Numerator / denominator	Proportion
Theme A: Appreciation for motivational and culturally resonant content	50 / 90	55.6%
Theme B: Increased awareness of available health-support services	40 / 90	44.4%
Theme C: Sustained connection with home-country health-support team	35 / 90	38.9%
Key-informant rating: "favorable"	22 / 22	100%
Key-informant rating: "neutral"	0 / 22	0%
Key-informant rating: "unfavorable"	0 / 22	0%

Case 3: Overseas Site-Visit Program

Overseas site visits conducted jointly by the occupational physician and the OHN at international construction and manufacturing sites were evaluated through post-visit feedback questionnaires and semi-structured interviews with employees and project managers. Feedback was gathered from all eight project sites across the observation period.

Employees at visited sites reported that in-person health consultations were valued, particularly the opportunity to receive medical explanations in Japanese, their native language, which addressed an unmet need given the limited opportunities for native-language health discussions while stationed overseas. Employees also recalled specific content from group health lectures delivered during visits, indicating effective short-term knowledge transfer. During individual consultations, the OHN’s observational notes recorded that the large majority of consulted employees presented with calm, engaged demeanour and did not exhibit visible signs of acute psychological distress. We note that this is an observational impression by the program provider rather than an aggregate quantitative measurement and is consistent with, but does not by itself prove, the absence of acute mental-health crises at the visited sites during or immediately following the visit period.

Numerical indicators of program reception are summarized in Table 8. Where values are approximate, the source is explicitly noted.

**Table 8:** Numerical indicators of the reception of the OHN-led overseas site-visit program across the eight projects (Case 3) Values denoted with “≈” are approximations explicitly attributed to routine clinical records and field notes; values reported as exact ratios were extracted directly from the interview log or the project leadership log. OHN: occupational health nurse

Indicator	Numerator / denominator	Proportion / count
Employee recognition of the OHN-coordinated site-visit program (approximate; from routine on-site interview log and post-return health-examination notes)	Cumulative deployed employees (≈ 450 across 8 projects)	≈ > 90%
On-site project leadership requesting continuation of the annual circuit visits at the next cycle (exact)	8 / 8 project leaderships	100%
Employees reporting a strengthened sense of connection with the home-country team (approximate; from on-site interview log and subsequent home-country health examinations)	Cumulative deployed employees	≈ 95%
Key-informant rating "favorable" (project managers, senior site supervisors, and safety-and-health representatives) (exact)	22 / 22 informants	100%
Written response rate to the ≈ 100-page post-visit report sent to corporate leadership (approximate; from feedback log)	Senior recipients at the head office	≈ 30%

Summary of outcome evaluation

Taken together across the three illustrative cases, the three operationally defined objective indicators were met within the limits of the indicators themselves: The Case 1 emergency aeromedical repatriation resulted in safe transport without an in-transit medical event, full clinical recovery, return to pre-event duties, and no documented residual occupational health consequences within the 12-month follow-up window. The Case 2 newsletter program elicited 90 returned questionnaires (response rate ≈ 20%) with the three pre-specified themes endorsed in 55.6%, 44.4%, and 38.9% of responses, respectively; and across the eight projects, no acute mental health event meeting the operational definition (requiring emergency aeromedical evacuation, involuntary admission, or active suicide prevention intervention) was documented within the project observation windows other than the Case 1 event. Semi-quantitative key informant appraisals were uniformly favorable: all 22/22 (100%) interviewed informants categorized the programs as “favorable,” and all 8/8 (100%) project leaderships requested continuation of the annual circuit visit program. Observational affect assessments by the OHN during on-site consultations indicated that the large majority of consulted employees presented with calm, engaged demeanour without visible distress.

We emphasize the following interpretive cautions, which are developed further in the Discussion and Limitations sections. First, key informants were purposively selected from project personnel who had directly observed or received the services and were interviewed by the program providers themselves; the resulting 100% favorable rating cannot be interpreted as comparative evidence of effectiveness and is most appropriately read as evidence of acceptability among engaged informants. Second, observational affect assessments by the OHN reflect clinical impressions recorded during consultations rather than validated psychometric measurements and are subject to observer bias. Third, the absence of documented acute crises during the observation period is consistent with, but does not by itself prove, prevention by the program; alternative explanations include selection of resilient employees for overseas assignment, general corporate culture, the relatively young and physically screened deployed population, and possible under-detection. Fourth, several reported aggregate proportions (≈>90% recognition, ≈95% connection) are approximations from routine records and field notes rather than from a formal sampling frame. With these cautions in mind, the convergent pattern of favourable acceptability and feasibility across all three cases, all eight projects, and multiple evaluation modes supports the practical utility and feasibility of this approach in the specific operational context described, but does not support claims of demonstrated clinical effectiveness.

## Discussion

The experiences described in this paper illuminate several practically relevant dimensions of occupational health support for overseas employees, with particular relevance to the role of OHNs as multidisciplinary team coordinators. We discuss the principal findings under five headings, then situate these findings within the broader global occupational health literature, examine the limits of generalizability, and explicitly weigh what the data can and cannot support.

The OHN as a coordinator: lessons from emergency psychiatric repatriation

Case 1 highlights a critical but underappreciated aspect of corporate mental health preparedness: the difficulty of securing acute psychiatric hospital admission following emergency repatriation. Repatriation for psychiatric reasons is a particularly complex undertaking, often requiring medical escort by suitably trained mental health professionals and pre-flight stabilisation of the patient’s mental state before aeromedical evacuation [20]. Even with the support of medical assistance service companies, arranging immediate inpatient psychiatric care for repatriated employees can be extremely challenging in the absence of pre-established institutional relationships. Our team structure addresses this vulnerability by including a psychiatrist who maintains affiliations with hospitals capable of emergency psychiatric admission, thereby creating a pre-established pathway from overseas crisis to domestic treatment. We propose that this structural consideration warrants attention when organizations assemble internal health support teams; we do not, however, claim that our specific model is the only or the best way to address this need.

The OHN’s role as the first responder and care coordinator in such situations appeared, in our experience, to be a key practical enabler of timely action. Their pre-existing relationship with the employee, knowledge of the employee’s medical and occupational history, and established communication channels with the psychiatrist enabled a response speed and continuity of care that would have been difficult to assemble de novo at the moment of crisis. Pre-deployment mental health screening, as recommended by researchers and practitioners in expatriate psychology, may further improve early identification of at-risk individuals [8,21].

Value of culturally sensitive health communication

Cases 2 and 3 illustrate the practical value of health support activities that extend beyond clinical intervention. The qualitative feedback from overseas employees who received the health newsletter (90 returned questionnaires comprising 50 appreciation-themed responses, 40 awareness-themed responses, and 35 connection-themed responses) and the favourable acceptability ratings of the site visit program suggest that proactive, multilingual, culturally resonant health communication and face-to-face on-site contact were well received in this setting. Research suggests that organizational strategies fostering a supportive work culture, rather than focusing exclusively on individual-level interventions, are central to protecting the mental health of workers in challenging environments [22]. The WHO guidelines on mental health at work similarly emphasize the importance of both organizational and individual interventions for comprehensive workplace mental-health management [2].

We hypothesize, but cannot demonstrate from these data, that the cumulative effect of these activities lowers barriers to employees seeking consultation with occupational health professionals and thereby strengthens the preventive dimension of the health support system. Such an effect would be particularly significant for expatriate populations, among whom stigma associated with mental health help-seeking remains a substantial barrier [9,23]. Practitioner-oriented reviews of expatriate-employee health have documented a global rise in mental health-related claims over the past decade, further underscoring the relevance of proactive outreach [9].

Mental health vulnerabilities of overseas employees

Employees working overseas, particularly those stationed in developing countries, face elevated psychological risk due to multiple concurrent stressors. Foyle and colleagues demonstrated a high incidence of affective and adjustment disorders among expatriates, significantly correlated with occupational anxiety, home country separation, acculturation challenges, and physical ill-health [7]. Truman and colleagues reported that expatriates experience higher rates of depression, sleep difficulties, anxiety, and attention deficits than domestic workers [10]. More recent evidence from international construction projects during the COVID-19 pandemic has further shown that the mental health of expatriates depends not only on individual psychological resources but also on team-level and organizational-level support [11]. Public health surveillance data on healthcare workers have highlighted that occupational psychosocial risk factors, such as harassment and burnout, compound the already-elevated baseline stressors of overseas deployment [24]. These findings underscore the relevance of comprehensive, organization-driven support systems rather than reliance solely on individual resilience, a point reinforced by the WHO’s recommendation that organizational interventions should be the primary approach to managing psychosocial risks in the workplace [2].

The overseas site-visit system: face-to-face support in the digital age

Our approach includes a systematic program of overseas site visits in which the OHN accompanies the occupational physician. During these visits, the OHN observes local healthcare conditions, gains firsthand experience managing anticipated medical situations, and conducts individual health consultations with employees alongside the physician. In our experience, this combination supports the team’s capacity for the early detection of health concerns and timely escalation, although we cannot quantitatively demonstrate this benefit from the data presented here.

Evidence from comparable programs is broadly supportive of this kind of approach. Comprehensive interventions for internationally deployed healthcare workers, incorporating cultural adaptation training, local healthcare system orientation, and mental health support, including family involvement, have been associated with reductions in psychological distress, increases in occupational health consultation rates, and decreased emergency repatriation cases [25]. While remote consultation methods that expanded during and after the COVID-19 pandemic have become widespread and offer practical advantages in accessibility and cost, in our setting, face-to-face consultations at overseas locations were considered to remain valuable for several reasons. First, the shared experience of the local environment provides contextual understanding that is difficult to replicate remotely. Second, individual consultations in employees’ native language address a significant unmet need, particularly for Japanese employees who have limited opportunities to discuss complex health matters in Japanese while stationed overseas. Third, a direct observation of the workplace and living environment enables a comprehensive assessment that combines clinical, environmental, and psychosocial dimensions [16,26]. Systematic reviews of evidence-based interventions for mental health in healthcare workers have catalogued more than 100 distinct interventions, the majority of which demonstrated significant positive changes in stress, anxiety, and burnout outcomes [13].

A three-tiered framework for OHN support of overseas employees

Drawing on our experience and the existing literature, we propose, as a heuristic for practitioners, that OHNs serving overseas-employee populations may usefully function within a three-tiered support framework: (a) bridging to the local healthcare system by establishing knowledge of and connections with local medical resources; (b) providing comprehensive life support, including attention to family members and daily living conditions; and (c) supporting career continuity by facilitating return-to-work processes following health-related interruptions and maintaining ongoing career-relevant health engagement. This three-tiered framework implies an expanded role for OHNs that extends beyond traditional clinical duties, encompassing functions as cultural mediators, life support coordinators, and crisis coordination liaisons [14,27]. Such an expanded role aligns with the broader evolution of occupational health nursing from a focus on workplace safety toward a comprehensive, well-being-oriented practice [15] and with the contemporary biopsychosocial paradigm articulated in ISO 45003:2021 and the WHO 2022 guidelines [2,16]. We offer this framework as a practical heuristic developed from our experience rather than as a validated model.

Essential qualities for OHNs in overseas employee support

Based on our experience, we tentatively identify the following qualities as practically valuable for OHNs tasked with coordinating mental health support for overseas employees: frequent interaction opportunities with employees to maintain “line-like” engagement; agility in responding promptly and empathetically to employee inquiries; a value system prioritizing kindness, empathy, and rapid response; experience accompanying occupational physicians on both domestic and international medical rounds; exposure to psychiatric consultations to develop practical patient management knowledge; and an awareness of emergency nursing principles with a broad understanding of emergency response procedures and communication protocols. These qualities are broadly consistent with international occupational health nursing competency frameworks [14,15]. Cultural competency training is also relevant, as culturally sensitive approaches have been shown to improve mental health outcomes in diverse and migrant populations [28]. We note, however, that this list of qualities is derived from a single-team experience and would benefit from validation through structured competency studies in additional settings.

Digital tools and future directions

Looking ahead, the integration of digital mental health tools into the overseas support framework represents a promising direction. Workplace digital mental health interventions (including online therapy platforms, stress management applications, and asynchronous digital counselling services) have been increasingly adopted to extend mental health support across geographical boundaries [29]. OHNs may play a useful intermediary role in leveraging these digital tools, maintaining the human-connection element while expanding the reach and availability of services. The telemedicine infrastructure developed since the COVID-19 pandemic has made hybrid approaches, combining periodic face-to-face site visits with ongoing remote support, increasingly feasible and sustainable [18]. The joint WHO-ILO policy brief on mental health at work provides a practical framework for implementing such integrated approaches at the national and organizational levels [18].

Post-visit follow-up and continuous support

Post-visit follow-up appeared, in our experience, to be valuable for sustaining the supportive relationship between the OHN and the employee. In our program, every employee identified during a site visit as potentially at risk of developing a mental-health issue received multiple rounds of structured, native-language email correspondence from the home country until the concern was resolved. Such personalized “health check-in” communications from the OHN can serve as a practical tool for maintaining engagement and facilitating early escalation when needed.

Situating the findings within the Global Occupational Health Agenda

The contribution of this report is best understood as one practice-based attempt to operationalize the integrated psychosocial paradigm of contemporary occupational health-articulated, among other instruments, in ISO 45003:2021 and the 2022 WHO guidelines [2,16], in the demanding context of overseas plant construction. The continuous “line-like” engagement model we describe is consistent with, and offered as a concrete operational instantiation of, the organizational-level interventions that current international guidance prioritizes; it is not offered as a substitute for the prospective, controlled effectiveness evidence that the field still requires.

Critical examination of limitations and their implications

We have already addressed several limitations in passing. We now examine them more critically and explicitly so that the boundaries of the evidence are unambiguous.

First, the study design is descriptive and retrospective rather than experimental, relying on qualitative observations and semi-quantitative retrospective appraisals rather than prospective quantitative outcome data or controlled comparisons. There is no prospective protocol, no control or comparison group, no randomization, and no pre/post-comparison on the full deployed population using validated instruments. As such, causal inferences about the effectiveness of specific interventions cannot be drawn. The convergent favorable findings reported here are entirely consistent with the program being beneficial; they are also consistent with the program being neutral and the favourable observations reflecting selection of resilient employees, the organization’s broader supportive culture, statistical fluctuation, or under-detection of adverse outcomes.

Second, the experiences described derive from a single organization - a Japan-headquartered multinational engineering organization - and may not be directly generalizable to other industries, organizational sizes, national or industry cultures, or workforce compositions. Cultural factors specific to the Japanese workplace context, such as the value placed on group harmony and corporate loyalty, the established norm of long employment tenure, and historically high stigma surrounding mental health help-seeking, may influence both the design choices of the support program and the way employees and managers responded to it. The transferability of this model to expatriate workforces of different national, industrial, or organizational backgrounds, and especially to small- and medium-sized enterprises that lack in-house multidisciplinary teams, is therefore uncertain.

Third, case selection was purposive: cases were drawn from projects in which the authors’ occupational health team had direct, intensive involvement. As discussed under Methods, the probable direction of this selection bias is toward favourable outcomes. Projects in which intensive health support was logistically infeasible, short-duration projects, projects with restricted worksite access, projects without an on-site safety-and-health structure, are systematically absent from this report and might plausibly have experienced different and in some respects worse trajectories. The findings should therefore be interpreted as illustrative of what an OHN-coordinated framework can look like when fully resourced rather than as representative of overseas projects in general. The contextual comparison provided in the Methods section is not a substitute for a quantitative comparison of included and non-included engagements; we have refrained from such a quantitative comparison because the underlying records for non-included engagements are heterogeneous and incomplete, and any constructed comparison would itself be vulnerable to the same documentation asymmetries that motivated the present caveat. Readers are accordingly encouraged to treat the cases reported here as upper-bound illustrations of what is possible when structural enabling conditions are in place, rather than as a basis for inference about overseas-deployment work in general.

Fourth, the case presentations involve a small number of illustrative examples drawn from a limited universe of eligible projects. Specifically, the absolute denominators involved are small by epidemiological standards: eight plant-construction projects; a cumulative population of 450 home-country-dispatched Japanese technical employees over 10 fiscal years; 90 newsletter-feedback respondents; and 22 interviewed key informants. These sample sizes preclude inferential statistical testing of effectiveness and limit the precision of any descriptive proportions reported. The findings should be read as exploratory and hypothesis-generating, not as statistically powered evidence of clinical efficacy.

Fifth, the semi-quantitative key informant appraisals were obtained from a purposively selected subset of project personnel rather than from every individual who received or observed the interventions and were collected by interviewers from the same team that delivered the program. The use of a categorical scale (“unfavorable,” “neutral,” “favorable”) rather than a validated numerical instrument limits precision; the provider-administered interview context introduces a real risk of acquiescence and courtesy bias. The 100% favorable rating across 22 informants should accordingly be read as evidence of acceptability among engaged informants, not as evidence of effectiveness.

Sixth, the observational affect assessments performed by the OHN, while consistent with established clinical nursing practice, do not constitute validated psychometric measurements. They are subject to observer bias, including a plausible positive bias arising from the OHN’s simultaneous role as program designer, and are reported here as supportive longitudinal clinical impressions rather than as aggregate quantitative outcomes.

Seventh, several aggregate proportions (employee recognition ≈> 90%; strengthened-connection ≈ 95%; written response rate ≈ 30%) are approximations drawn from routine clinical records and field notes rather than from a formal sampling frame; we have flagged these explicitly and have not used them to support any inferential claim. We acknowledge openly that, despite the strengthened reporting of data sources and derivation procedures provided in the Methods section, a residual degree of imprecision is intrinsic to a retrospective analysis of routinely collected practice records, and the approximated proportions reported here should accordingly be read as broad-magnitude indicators of program reception rather than as point estimates from a formal sampling frame. Where possible, exact numerators and denominators have been substituted (Tables 6-8), and the data source for each remaining approximation is named both at the point of reporting and in the relevant table footnote, so that readers can independently judge the weight to attach to each value.

Eighth, the absence of validated mental-health outcome instruments, such as the PHQ-9 for depression, the GAD-7 for anxiety, or the WHO-5 Well-Being Index, means that we cannot report standardized symptom scores, pre/post-change scores, or population-level prevalence estimates for the deployed cohort. The crude binary objective indicators we did use (safe repatriation, absence of residual harm, absence of acute crises) are appropriate within their operational definitions but capture only a narrow slice of the mental-health construct.

Ninth, qualitative findings are presented in summary form rather than through verbatim direct quotations from employees and managers. We have not included direct quotes because, as a practice-based report drawing on routine occupational-health interactions and on highly sensitive mental-health consultations, the relevant exchanges were not audio-recorded or transcribed verbatim, and our overriding obligation of medical confidentiality precludes the reconstruction of identifiable utterances even in anonymized form. We have endeavored, instead, to convey the general atmosphere and the recurring concerns through descriptive synthesis.

Finally, this report does not deeply analyze near-misses, partial failures, or the specific circumstances under which the framework did not perform as intended. We acknowledge that this asymmetry reduces balance and that future reports from our team and from others would benefit from explicit attention to such cases.

Future studies should employ prospective designs with validated outcome measures, larger and more systematically selected samples, multi-organizational and multi-industry comparisons, comparison or control groups where ethically and operationally feasible, and explicit examination of cases where the model did not work, in order to generate more robust evidence regarding the effectiveness of OHN-coordinated mental health support frameworks for overseas employees.

## Conclusions

Our practice experience suggests that positioning OHNs as coordinators within a multidisciplinary team is a feasible and well-accepted approach to supporting the mental health of Japanese technical employees on overseas plant construction assignments. Site visits, regular multilingual communication, and pre-established psychiatric referral pathways appeared practically valuable in this setting. Continuous, “line-like” engagement by the OHN, as opposed to isolated, episodic interactions, appeared, in our experience, to support early identification of concerns and to facilitate timely escalation when needed.

Given the retrospective, descriptive, single-organization design, the small number of projects and informants, the absence of validated outcome measures, and the absence of a comparison group, these findings should be interpreted as evidence of feasibility and acceptability within a specific operational context rather than as proof of clinical effectiveness, comparative superiority, or generalizable benefit. We deliberately refrain from recommending that organizations “must” adopt this specific model, or from describing the OHN coordinator role as “indispensable” in the abstract; what we can say is that, in this organization, this team configuration was operationally workable and was well received by the employees and managers who engaged with it.

Further prospective studies with validated outcome measures, multi-organizational and multi-industry sampling, and pre/post or comparison-group designs are needed to evaluate the effectiveness of this coordinator model and to test whether it can be transferred to other industries, organizational sizes, and cultural contexts. Practitioners considering analogous frameworks may find the operational details reported here, particularly the structured combination of on-site face-to-face consultation, structured email follow-up, multilingual newsletter communication, and pre-established psychiatric referral, useful as a starting point for adaptation to their own settings, while bearing in mind the limits of the evidence reported here.

## Figures and Tables

**Figure 1:**
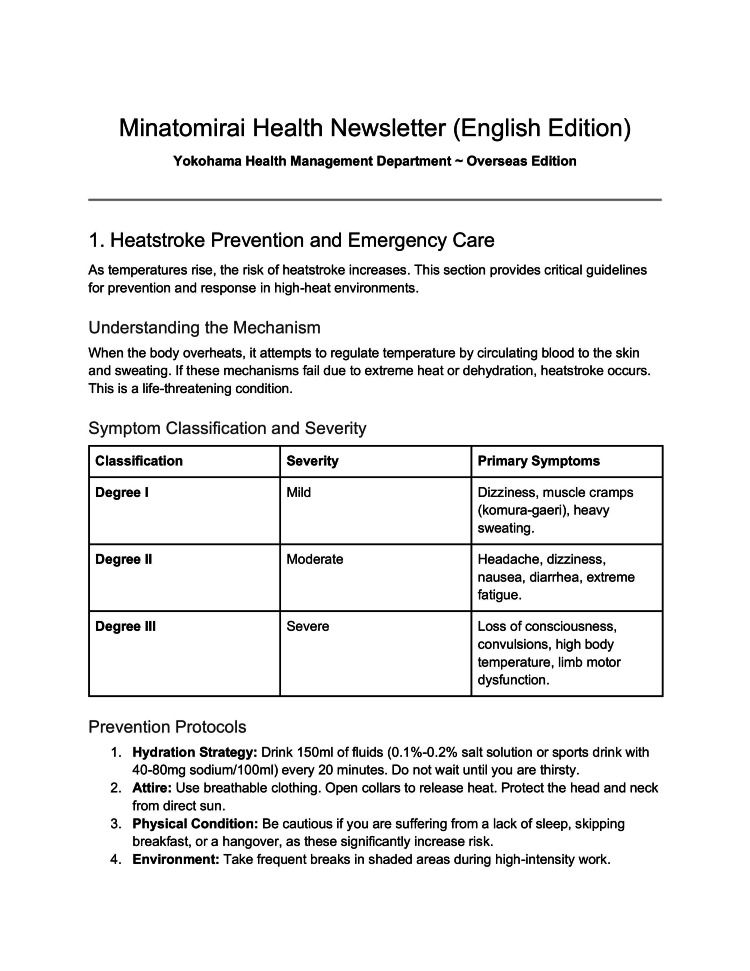
Heatstroke prevention and emergency care for overseas work environments Representative page of the Minatomirai Health Newsletter (English edition), page 1: heatstroke prevention and emergency care, including a severity classification and practical prevention and response protocols tailored to overseas work environments.

**Figure 2:**
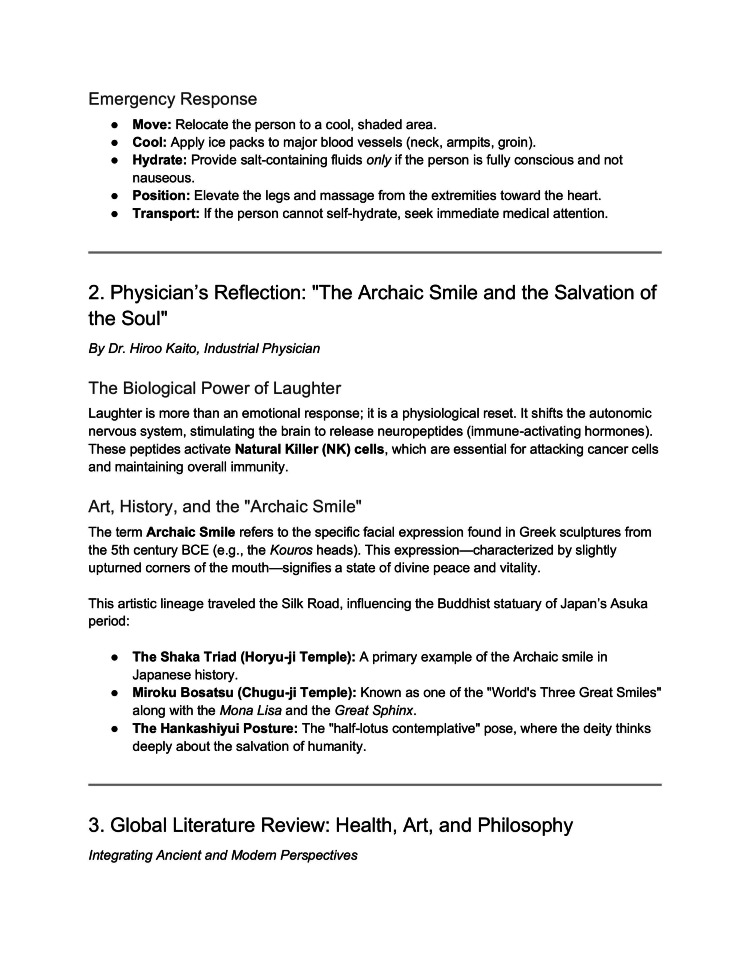
Emergency response protocols and a reflective essay on laughter and the archaic smile Representative page of the Minatomirai Health Newsletter (English edition), page 2: emergency response protocols and the physician’s reflective essay on the biological power of laughter and the “Archaic Smile,” illustrating the integration of medical and culturally resonant content.

**Figure 3:**
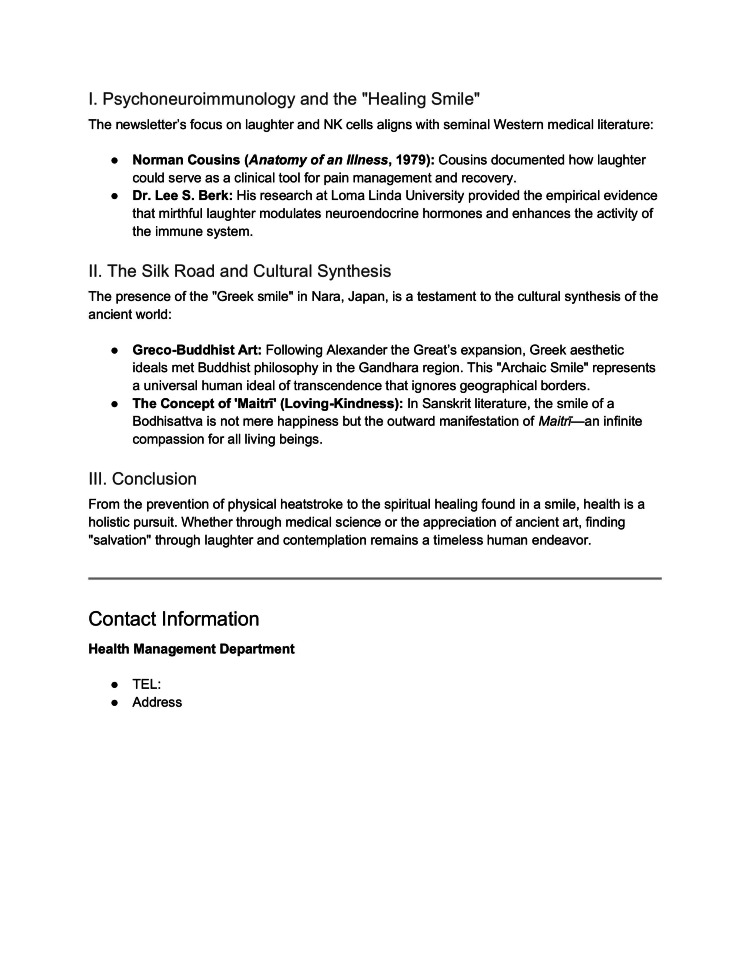
Psychoneuroimmunology and humanistic reflection in a cross‑cultural health context Representative page of the Minatomirai Health Newsletter (English edition), page 3: psychoneuroimmunology and humanistic reflection, demonstrating the editorial integration of biomedical evidence with humanistic and cross-cultural perspectives.

## Appendices

Representative pages of the Minatomirai Health Newsletter

Overview

Appendices A-C present representative pages of the Minatomirai Health Newsletter (English edition), the multilingual monthly health communication tool referenced in Case 2. The newsletter was distributed electronically to all 450 deployed employees across the eight project sites throughout the observation period. The three pages shown together constitute one representative monthly issue.

Editorial Design

Each issue integrates three content elements: (i) practical medical guidance addressing occupational health risks encountered at overseas construction and industrial sites, (ii) reflective essays by the occupational physician that connect biomedical topics to broader cultural and philosophical themes, and (iii) short literature reviews integrating scientific, artistic, and cross-cultural perspectives.

This composition is designed to deliver actionable health information while sustaining the emotional and intellectual engagement of employees working far from their home support systems.

Employee Feedback (Summary)

As summarized in the Results section, employee feedback indicated that the motivational and integrative design of the newsletter was favorably received. Key informants, including project managers and senior staff, likewise evaluated the program favorably, with the interpretive cautions described in the main text.

Appendix A

Appendix B 

Appendix C 

## References

[1] Dewa CS, Loong D, Bonato S, Thanh NX, Jacobs P (2014). How does burnout affect physician productivity? A systematic literature review. BMC Health Serv Res.

[2] (2026). World Health Organization. WHO guidelines on mental health at work. https://www.who.int/publications/i/item/9789240053052.

[3] Dewa CS, Loong D, Bonato S, Joosen MC (2015). The effectiveness of return-to-work interventions that incorporate work-focused problem-solving skills for workers with sickness absences related to mental disorders: a systematic literature review. BMJ Open.

[4] Ramazzini B (1940). De Morbis Artificum Diatriba [Diseases of Workers]. Modena: Antonio Capponi; 1700. (English translation: Wright WC, transl.

[5] Hamilton A (1943). Exploring the Dangerous Trades: The Autobiography of Alice Hamilton. M.D. Boston.

[6] Felton JS (1985). The genesis of American occupational health nursing. Part I. Occup Health Nurs.

[7] Foyle MF, Beer MD, Watson JP (1998). Expatriate mental health. Acta Psychiatr Scand.

[8] Filipič Sterle M, Vervoort T, Verhofstadt LL (2018). Social support, adjustment, and psychological distress of help-seeking expatriates. Psychol Belg.

[9] Patel M (2026). Expat mental health: taking a preventative approach. Relocate Magazine.

[10] Truman S, Sharar D, Pompe J (2011). The mental health status of expatriate versus U.S. domestic workers: a comparative study. Int J Ment Health.

[11] Gao L, Deng X, Yang W, Fang J (2022). COVID-19 related stressors and mental health outcomes of expatriates in international construction. Front Public Health.

[12] Aegerter KH, Meyer AH, Gaab J, Ooi YP (2025). Expatriation stressors and the well-being of accompanying partners: a commonality analysis approach. Front Psychol.

[13] Anger WK, Dimoff JK, Alley L (2024). Addressing health care workers’ mental health: a systematic review of evidence-based interventions and current resources. Am J Public Health.

[14] (2026). American Association of Occupational Health Nurses. What is occupational and environmental health nursing?. https://www.aaohn.org/About/What-is-Occupational-and-Environmental-Health-Nursing.

[15] Lozano-Lorca M, Olmedo-Requena R, Vega-Galindo MV (2026). The mental health and wellbeing of nurses and midwives in the United Kingdom. Int J Environ Res Public Health.

[16] (2026). International Organization for Standardization. ISO 45003: 2021. Occupational health and safety management — Psychological health and safety at work — Guidelines for managing psychosocial risks. https://www.iso.org/standard/64283.html.

[17] (2026). Federation of Occupational Health Nurses within the European Union: a core curriculum for occupational health nursing in Europe. FOHNEU.

[18] (2026). World Health Organization, International Labour Organization. https://www.who.int/publications/i/item/9789240057944.

[19] Braun V, Clarke V (2006). Using thematic analysis in psychology. Qual Res Psychol.

[20] Flaherty G, Chai SY, Hallahan B (2021). To travel is to live: embracing the emerging field of travel psychiatry. BJPsych Bull.

[21] Fox DJ (2026). Expatriate mental-health challenges. Psychology Today.

[22] Maple JL, Willis K, Lewis S (2024). Healthcare workers’ perceptions of strategies supportive of their mental health. J Med Surg Public Health.

[23] Thornicroft G, Sunkel C, Alikhon Aliev A (2022). The Lancet Commission on ending stigma and discrimination in mental health. Lancet.

[24] Nigam JA, Barker RM, Cunningham TR, Swanson NG, Chosewood LC (2026). Vital signs: health worker-perceived working conditions and symptoms of poor mental health - Quality of Worklife Survey, United States, 2018-2022. MMWR Morb Mortal Wkly Rep.

[25] (2026). Greater Manchester Mental Health NHS Foundation Trust. International nurse programme. Manchester: GMMH.

[26] Bhugra D, Watson C, Wijesuriya R (2021). Culture and mental health: a comprehensive textbook. Br J Psych Int.

[27] Hayes J, Schimel J, Arndt J, Faucher EH (2010). A theoretical and empirical review of the death-thought accessibility concept in terror management research. Psychol Bull.

[28] Bhui K, Warfa N, Edonya P, McKenzie K, Bhugra D (2007). Cultural competence in mental health care: a review of model evaluations. BMC Health Serv Res.

[29] Phillips EA, Gordeev VS, Schreyögg J (2019). Effectiveness of occupational e-mental health interventions: a systematic review and meta-analysis of randomized controlled trials. Scand J Work Environ Health.

